# Supplementary material for: A building-scale modeling framework for urban net-zero transitions in Nanjing
Source: Nat Commun. 2025 Oct 8;16:8954. doi: 10.1038/s41467-025-64016-7 (PMC12508441; doi:10.1038/s41467-025-64016-7)
Supplement: Supplementary file 1 — Supplementary Information [file 41467_2025_64016_MOESM1_ESM.pdf]

Supplementary Information

A building-scale modeling framework for urban net-zero transitions in Nanjing

Yuxin Chen[1], Zhenyu Wang[1], Quan Wen[1], Jing Meng[2], Jingwen Huo[2], Shuping Li[1], Li Zhou[3], Peipei Chen[4], Diling Liang[2], Jun Bi[5], Dabo Guan[1 *]

Affiliations:

1. Department of Earth System Science, Tsinghua University, Beijing 100084, China.
2. The Bartlett School of Sustainable Construction, University College London, London WC1E 7HB, UK.
3. China Renewable Energy Engineering Institute, Beijing 100011, China.
4. Cambridge Judge Business School, University of Cambridge, Cambridge CB1 1TN, UK.
5. School of the Environment, Nanjing University, Jiangsu 210023, China.

*Corresponding Author. Email: **guandabo@tsinghua.edu.cn**

Supplementary Figures

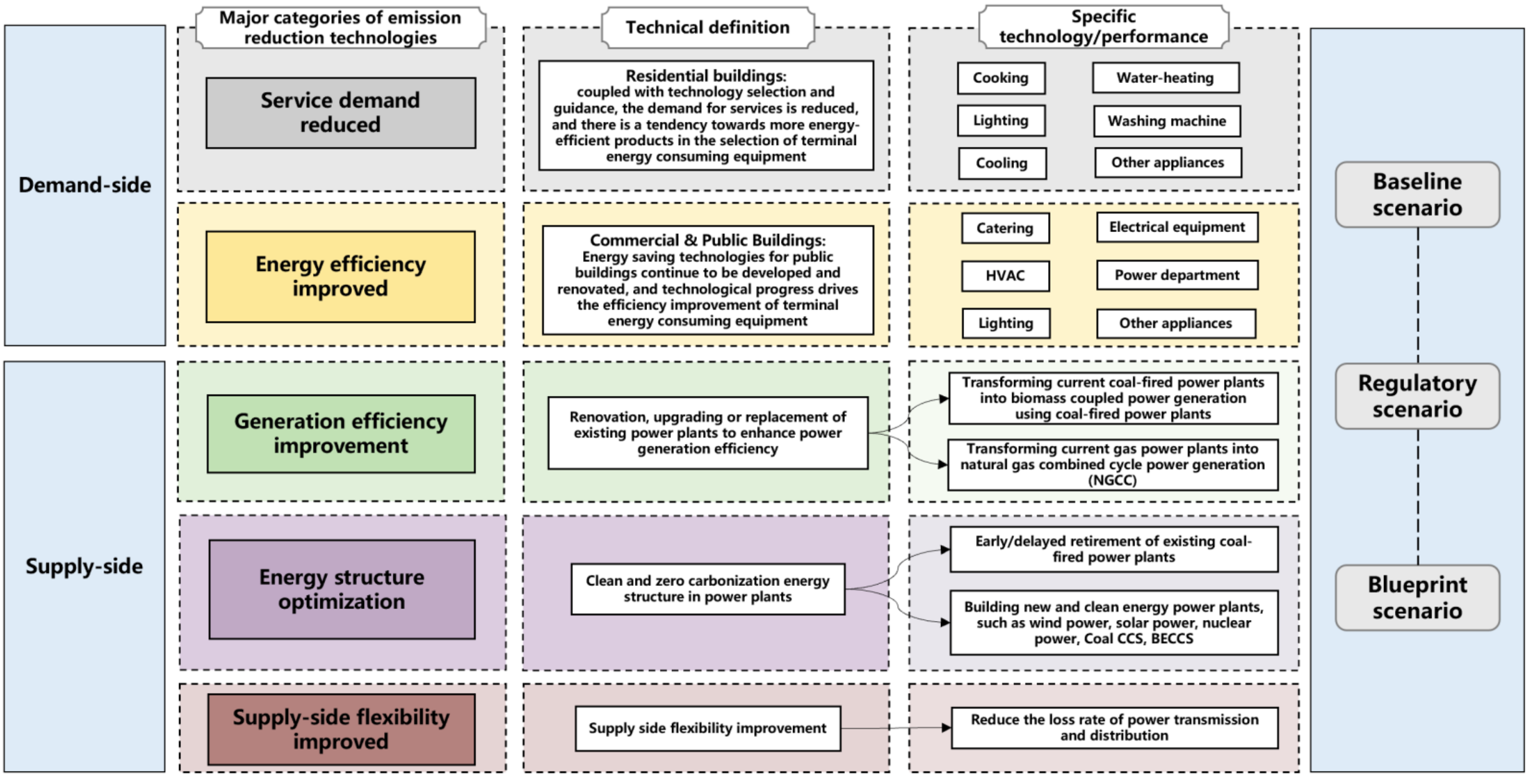

Supplementary Fig.1 | Pathways for future emission reduction technologies and scenario framework during the operational phase of buildings in Nanjing. The analysis classifies mitigation technologies through demand-side (service demand reduction and end-use equipment efficiency enhancement) and supply-side (power generation efficiency improvements, energy structure optimization, and load flexibility enhancement) approaches, simulating emission trajectories under three distinct technological configurations: baseline, regulatory, and blueprint scenarios.

Basing on the CEADs-building model, we combine a series of macro- and micro-level parameters to assess the contributions of various emission reduction technologies under three scenarios: baseline, regulatory, and blueprint. Each scenario incorporates both demand-side and supply-side CO_2 emission reduction strategies. Given that China has the largest coal power infrastructure globally, achieving global net-zero emissions targets necessitates the rapid phasing out of coal-fired power plants within the next few decades. According to Cui et al. (2021), China's coal-fired power plants must be fully decommissioned by 2045 or 2055 to meet the climate goal of limiting temperature rise to 1.5°C or 2°C, respectively[1]. Currently, coal-fired power plants are the dominant energy source and the largest emitters in Nanjing. In this study, the regulatory scenario aligns with the 2°C temperature control target, involving the complete phase-out of coal-fired power plants by 2055. The blueprint scenario, aligned with the 1.5°C target, envisions an accelerated phase-out, with all coal-fired power plants retiring by 2045.

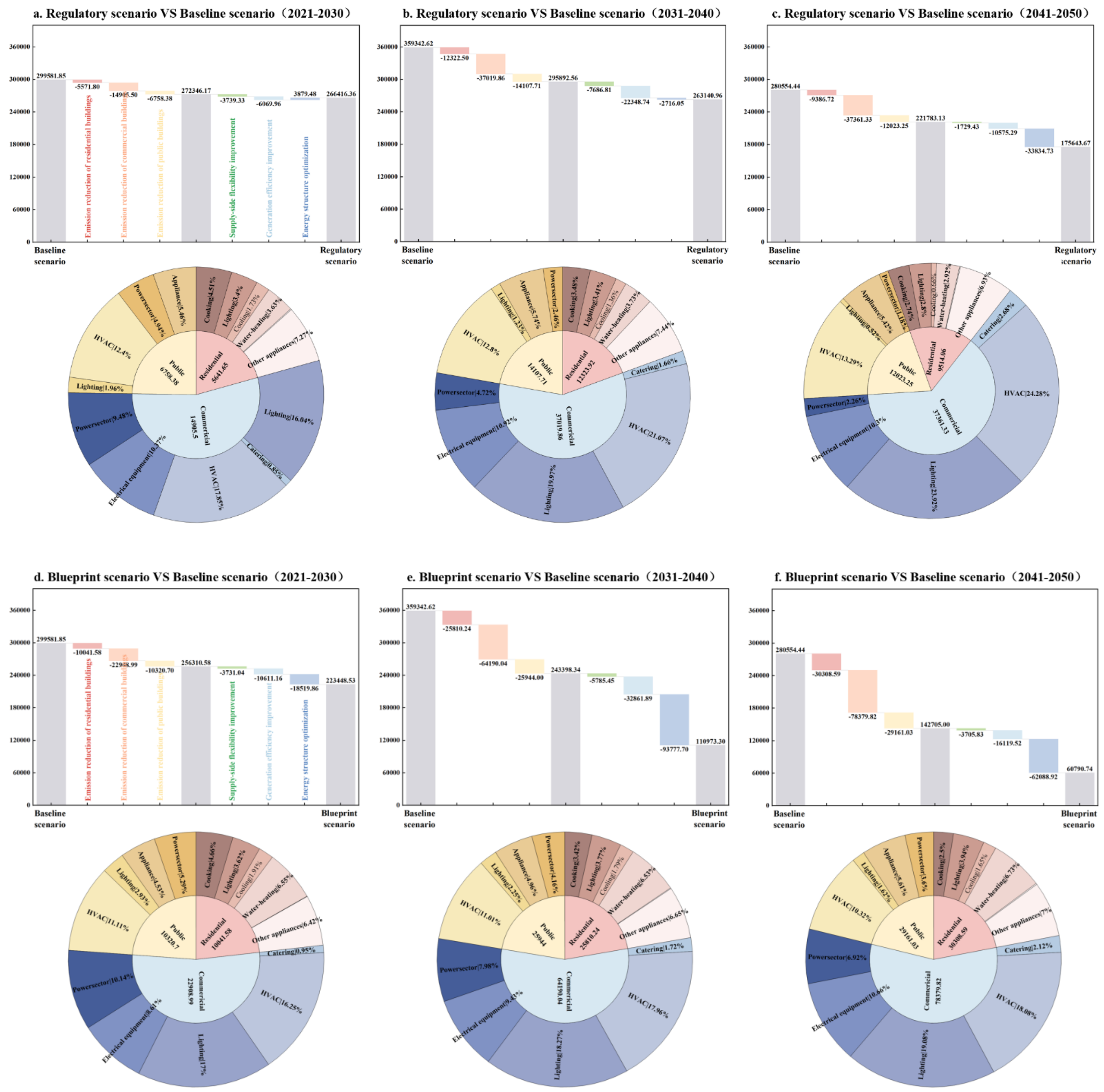

Supplementary Fig.2 | Comparison of cumulative emissions and technological contribution of each scenario under demand and supply side emission reduction technologies. 2a, **2b**, and **2c** present a comparison of cumulative CO_2 emissions resulting from energy consumption during the operational phase of buildings under the regulatory and baseline scenarios for the periods 2021-2030, 2031-2040, and 2041-2050. The waterfall charts illustrate the emission reduction technologies required to transition from the baseline to the regulatory scenario within each respective period, along with the contributions of each technology. The left half of the waterfall chart focuses on demand-side emission reduction measures, while the right half addresses supply-side measures. The sunrise plot highlights the CO_2 emission reduction potential for different

building types on the demand-side, as well as for various end-use energy devices within those building types. Similarly, **2d**, **2e**, and **2f** follow the same structure, but they compare the blueprint scenario with the baseline scenario.

In terms of demand-side emissions reduction, commercial buildings consistently demonstrate the highest absolute reductions across all time periods. Notably, the proportion of emissions reductions from commercial buildings is projected to be greater in 2041-2050 compared to 2021-2030 and 2031-2040, underscoring their substantial potential for emissions mitigation and the extended timeline required to achieve these reductions. Within this category, commercial HVAC systems, lighting, and electrical equipment are particularly significant contributors to emissions reductions. Specifically, improvements in energy efficiency and reductions in CO_2 emissions per unit of electricity consumption in the commercial HVAC sector have led to emissions reductions of 4,872.96, 13,369.44, and 14,298.41 kt CO_2e over the three decades, highlighting the critical role of HVAC systems in future energy-saving strategies. On the supply-side, the early retirement of coal-fired power plants plays the most significant role in reducing CO_2 emissions between 2041 and 2050. Phasing out outdated capacity as quickly as possible facilitates the adjustment of the energy mix and the deployment of more efficient power generation technologies. Additionally, during the 2031-2040 period, significant emissions reductions are achieved by enhancing the efficiency of coal-fired power plants, specifically through their conversion to biomass-coupled facilities and the transformation of traditional gas power plants into natural gas combined cycle (NGCC) plants. This approach accounts for 68.24% of the supply-side emissions reductions during this period.

The comparison of cumulative emissions between the blueprint scenario and the baseline scenario across three time periods is illustrated in Fig. 2d, 2e, and 2f. In the blueprint scenario, demand-side emission reductions play a particularly significant role compared to the regulatory scenario. For example, improvements in energy efficiency for commercial lighting in the blueprint scenario result in a cumulative emission reduction of 54,835.89 kt CO_2e from 2021 to 2050, whereas the regulatory scenario achieves a cumulative reduction of 31,137.49 kt CO_2e. This difference is likely due to the blueprint scenario's aggressive advancement of energy-saving technologies, which substantially reduces energy consumption intensity and CO_2 emissions in commercial and public buildings. From a supply-side perspective, under the 2°C target, the mandated retirement of all coal-fired power plants by 2045 leads to significant emission reduction impacts, particularly between 2031 and 2050. During this period, the transition from outdated to more efficient energy sources and the restructuring of the energy mix are projected to achieve a cumulative CO_2 emission reduction of 155,866.62 kt CO_2e.

Overall, the best practice for energy consumption and CO_2 emission reduction during the operation phase of buildings is for both the demand and supply sides to exert efforts simultaneously. It can be seen that the higher the CO_2 emission reduction standards and stronger the emission reduction technologies adopted by the demand and supply sides, the greater the CO_2 emission reduction amplitude. Therefore, high standards of demand-side management, early retirement of coal-fired power plants, and other supply-side emission reduction technologies are of great importance. In the blueprint scenario, coal-fired power plants are gradually retired before reaching 30 years of operation, and when combined with transformative CO_2 reduction technologies, this approach

emerges as the most effective strategy for minimizing CO_2 emissions associated with building operations.

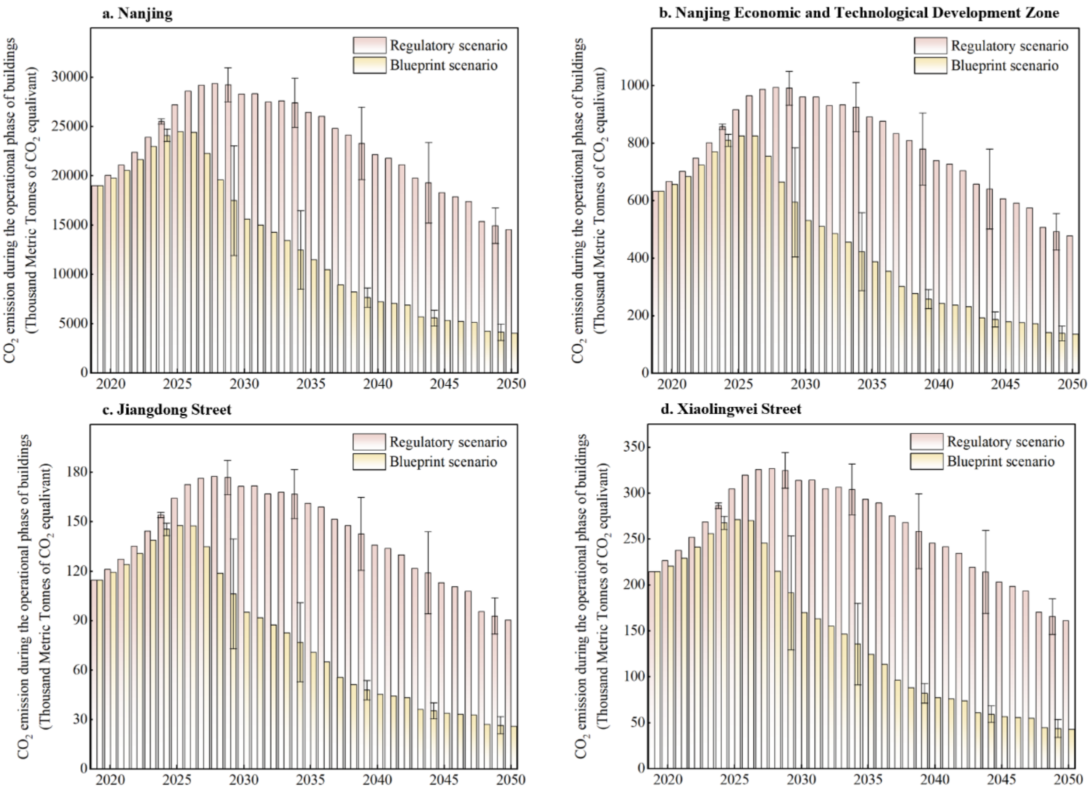

Supplementary Fig.3 | Uncertainty analysis of CO_2 emissions in different street during the operational phase of buildings. Parameter perturbations were performed on two key variables: energy intensity of HVAC systems (demand side) and decommissioning timeline of coal-fired plants (supply side). Column plots display emission scenarios with error bars denoting upper and lower bounds of new scenarios. Panel a shows city-wide results for Nanjing, while panels b-d present findings for Nanjing Economic and Technological Development Zone (b), Jiangdong street (c), and Xiaolingwei street (d), respectively.

Sensitivity analysis serves as a quantitative uncertainty assessment technique that evaluates the degree to which variations in key parameters influence critical indicators. This approach fundamentally examines trends in target metrics by systematically altering relevant variables to observe corresponding responses. In this study, we conducted a sensitivity analysis by perturbing two critical model parameters: (1) the energy intensity of HVAC systems (demand side) and (2) the retirement timeline of coal-fired power plants (supply side). This generated four updated scenarios (see Supplementary Table 1 for definitions), with results presented in Supplementary Fig.3. By adopting range-based outputs rather than single-point estimates, we reduced analytical rigidity and better characterized uncertainty.

Supplementary Table 1 | Definitions of the four updated scenarios

**Scenario Category**	**Updated Scenario**	**Key Parameter Selection**	**Parameter Variation**
Regulatory	Updated	Demand-side: HVAC energy intensity	Energy intensity increased

scenario	Scenario 1	Supply-side: Coal power plant retirement schedule	Retirement delayed by 5 years
	Updated	Demand-side: HVAC energy intensity	Energy intensity decreased
	Scenario 2	Supply-side: Coal power plant retirement schedule	Retirement advanced by 5 years
	Updated	Demand-side: HVAC energy intensity	Energy intensity increased
Blueprint	Scenario 3	Supply-side: Coal power plant retirement schedule	Retirement delayed by 5 years
scenario	Updated	Demand-side: HVAC energy intensity	Energy intensity decreased
	Scenario 4	Supply-side: Coal power plant retirement schedule	Retirement advanced by 5 years

The sensitivity analysis reveals significant findings under the regulatory scenario framework: (i) Scenario Update 1, incorporating elevated HVAC energy intensity and delayed plant retirements, demonstrates a 2-year postponement of Nanjing's operational CO_2 emissions peak accompanied by a 1.186 Mt CO_2 increase in peak magnitude; (ii) Conversely, Scenario Update 2, featuring reduced HVAC intensity and accelerated retirements, achieves 1-year earlier peaking with a 0.789 Mt CO_2 reduction in peak levels. These results quantitatively demonstrate that modifications to both HVAC energy intensity and coal plant retirement schedules can substantially alter both the temporal occurrence and quantitative magnitude of emission peaks. Similar response patterns were observed for the blueprint scenario and its corresponding Updates 3 and 4, confirming the robustness of these parameter-dependent relationships across multiple policy frameworks.

Supplementary Table 2 | Peak time and peak values under updated and reference scenarios

	Peak time (year)	Peak value (Mt CO_2)
Regulatory scenario	2029	29.930
Updated Scenario 1	2031	31.116
Updated Scenario 2	2028	29.141
Blueprint scenario	2026	25.928
Updated Scenario 3	2026	26.609
Updated Scenario 4	2024	23.383

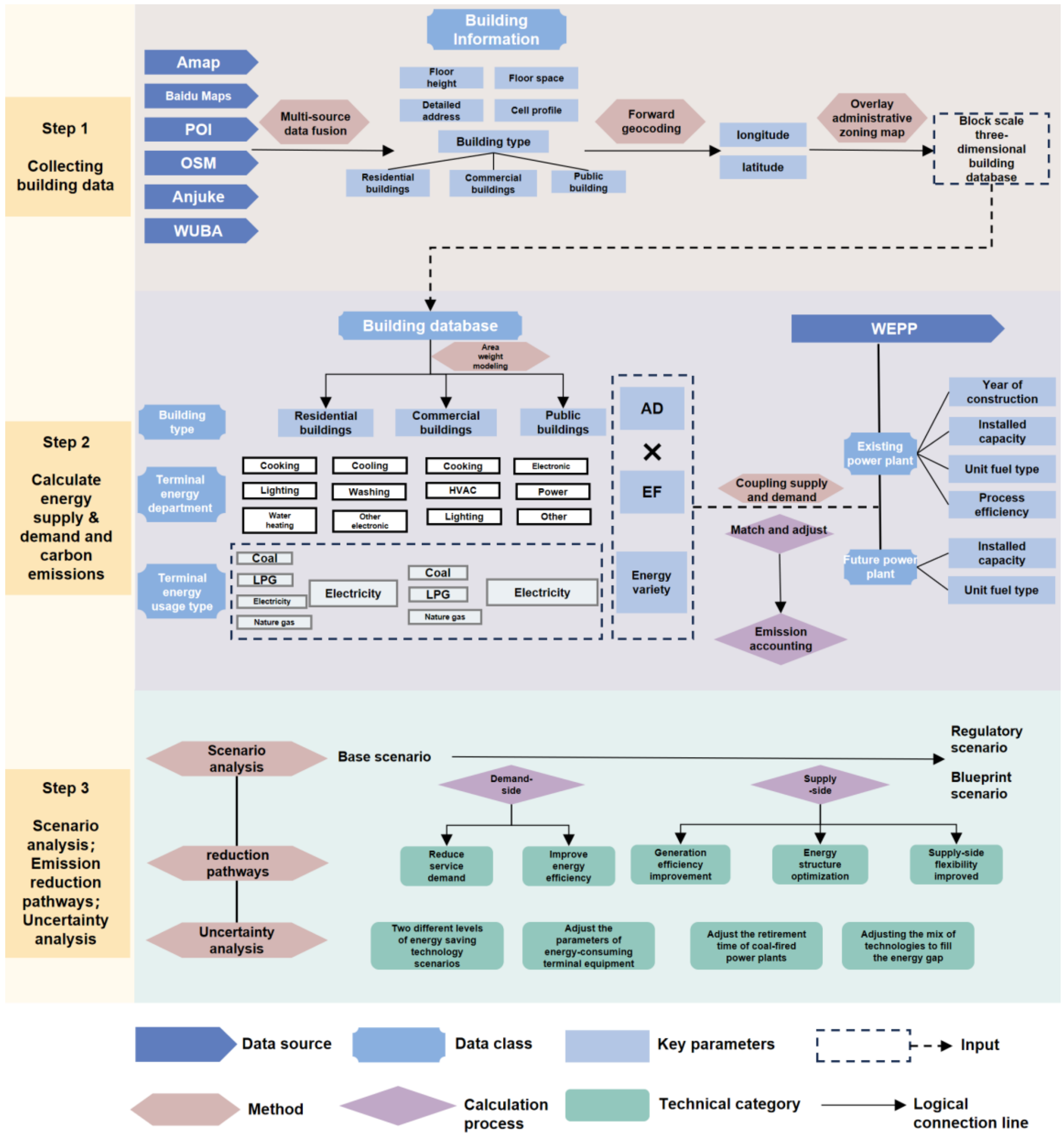

Supplementary Fig.4 | Schematic representation of the research framework. Research framework following a "data-assessment-pathway" methodology. (1) Data integration: Multi-source data fusion constructs a building-level database with multidimensional indicators. (2) Demand-supply coupling: Demand-driven modeling quantifies operational energy use and associated CO_2 emissions through integrated supply-side responses. (3) Pathway exploration: Scenario-based emission reduction analysis with uncertainty quantification, supported by technical workflow schematics.

Supplementary Tables

Supplementary Table 3 | Compendium of building decarbonization literature (representative studies)

Dimension	Technology	Typical research	Range	Period	Method
Demand-side	Building envelope	Ma et al.（2023）[2]	Urban architecture in northern China	2020-2060	MESSAGE
		Rodrigues et al.（2024）[3]	An office building in Portugal	——	LCA

	Renewable energy building renovation	Shi et al.（2016）[4]	China Construction Sector	2010-2050	China TIMES
		Aguacil et al.（2024）[5]	Typical residential buildings in Switzerland	——	LCA
	Heating and cooling system	Sarica et al.（2023）[6]	Turkish construction sector	2015-2050	TIMES
		Kong et al.（2012）[7]	China Construction Sector	——	Review
	Promotion of energy-saving and efficient household appliances	Delmastro et al.（2015）[8]	Chinese residential building	2030	EFB
	End use equipment electrification	Langevin et al.（2023）[9]	Residential and commercial buildings in US	2005-2050	Scout+GridSIM
Supply-side	Clean energy structure	Kannan & Strachan（2009）[10]	UK construction sector	2000-2050	TIMES
		Gambhir et al.（2013）[11]	China's industry, construction and transportation sectors	2010-2050	MESSAGE
	Improvement of district heating efficiency	McNeil et al.（2016）[12]	Urban residential and commercial buildings in China	2010-2030	LBNL
	Other disruptive technologies for decarbonization	Xiao et al.（2022）[13]	China Power Sector	2020-2030	SD-CGE
		Oliveira et al.（2016）[14]	Brazilian energy sector	2010-2050	MESSAGE

Supplementary Table 4 | Energy consumption level of cooking and other terminal energy use equipments in residential buildings

Scenario	Year	Final Energy Intensity（kgce/m2）				Share（%）			
		Coal	Natural Gas	LPG	Electricity	Coal	Natural Gas	LPG	Electricity
Base Year	2019	2.12	2.12	2.12	1.86	8.77	39.5	34.13	17.6
Baseline scenario	2030	1.95	1.95	1.95	1.25	7.75	43.75	29.75	18.75
	2060	0.99	0.99	0.99	0.35	4.53	50.36	5.06	40.05
Regulatory scenario	2030	1.87	1.87	1.87	0.95	6.75	48.75	25.75	18.75
	2060	0.87	0.87	0.87	0.25	4.007	51.87	4.19	40.03
Blueprint scenario	2030	1.78	1.78	1.78	0.75	5.75	51.75	23.75	18.75
	2060	0.77	0.77	0.77	0.15	3.254	53.38	3.35	40.02

Equipment	Year	Indicator	Scenario		
			Baseline scenario	Regulatory scenario	Blueprint scenario
Lighting	2019	Share(%)	100	100	100
		EI(kwh/m2)	5.1	5.1	5.1
	2050	Share(%)	100	100	100
		EI (kwh/m2)	7.3	6.1	5.3
Cooling	2019	Share(%)	100.75	100.75	100.75
		EI (kgce/m2)	0.3394	0.3394	0.3394
	2030	Share(%)	0.3374	0.3254	0.3174
		EI (kgce/m2)	115	110	107
	2040	Share(%)	0.3265	0.3155	0.3015

		EI (kgce/m2)		125	120	116
	2050	Share(%)		0.3250	0.3050	0.2900
		EI (kgce/m2)		132.5	127.5	123
Water heating	2019	Share(%)		100	100	100
		EI (kwh/m2)		3.9	3.9	3.9
	2050	Share(%)		100	100	100
		EI (kwh/m2)		10.4	9.1	6.2
Washing machine	2019	Share(%)	Drum	55	55	55
			Wave	45	45	45
		EI (tce/unit)	Drum	0.0147	0.0147	0.0147
			Wave	0.0024	0.0024	0.0024
	2050	Share(%)	Drum	27	24	15
			Wave	73	76	85
		EI (tce/unit)	Drum	0.015	0.016	0.02
			Wave	0.0025	0.0028	0.0035
Other Appliances	2019	Share(%)		100	100	100
		EI (kwh/m2)		7.3525	7.3525	7.3525
	2050	Share(%)		100	100	100
		EI (kwh/m2)		12.7	9.9	9

Supplementary Table 5 | Energy consumption level of catering and other terminal energy use equipments in commercial and public buildings

Scenario	Year	Share（%）			Final Energy Intensity（kgce/m2）		
		Coal	Natural Gas	LPG	Coal	Natural Gas	LPG
Base Year	2019	13.55	0.32	5.55	16.84	17.01	16.94
Baseline scenario	2030	10	2	4.5	16	16.2	16.2
	2040	9.5	2.5	4	15.5	15.8	15.7
	2060	4	3	3.5	14.5	15.1	15
Regulatory scenario	2030	9.85	3	4.45	15.5	16	16
	2040	8	4	3.85	14	15.5	15.6
	2060	3	5	3.05	13.5	14.6	14.5
Blueprint scenario	2030	9.5	3.5	4.4	15	15.85	15
	2040	6.5	5	3.65	13.5	15.2	15.4
	2060	2	5.5	3	13	14	14.2

Equipment	Year	Indicator	Scenario		
			Baseline scenario	Regulatory scenario	Blueprint scenario
HVAC	2019	Share(%)	100	100	100
		EI (kgce/m2)	42	42	42

	2050	Share(%)		100	100	100
		EI (kgce/m2)		42	25	18
Lighting	2019	Share(%)		100	100	100
		EI (kwh/m2)		16.6	16.6	16.6
	2050	Share(%)		100	100	100
		EI (kwh/m2)		43.6	24.5	20
Electrical equipment	2019	Share(%)		100	100	100
		EI (kwh/m2)		13.7725	13.7725	13.7725
	2050	Share(%)		100	100	100
		EI (kwh/m2)		37.1	29.8	27.9
Powersector	2019	Share(%)		100	100	100
		EI (kgce/m2)		2.56	2.56	2.56
	2030	Share(%)		100	100	100
		EI (kgce/m2)		2.16	1.87	1.65
	2040	Share(%)		100	100	100
		EI (kgce/m2)		1.73	1.64	1.07
	2050	Share(%)		100	100	100
		EI (kgce/m2)		1.36	1.08	0.11
Lighting-Public	2019	Share (%)	Ordinary	60	60	60
			Energy-saving	40	40	40
		EI (kgce/m2)	Ordinary	0.9	0.9	0.9
			Energy-saving	0.3	0.3	0.3
	2030	Share (%)	Ordinary	50	40	30
			Energy-saving	50	60	70
		EI (kgce/m2)	Ordinary	1	1	0.8
			Energy-saving	0.3	0.2	0.14
	2045	Share (%)	Ordinary	40	30	20
			Energy-saving	60	70	80
		EI (kgce/m2)	Ordinary	1.2	1.5	0.7
			Energy-saving	0.2	0.12	0.02
	2060	Share (%)	Ordinary	25	15	0
			Energy-saving	75	85	100
		EI (kgce/m2)	Ordinary	1.6	1.8	1
			Energy-saving	0.1	0.04	-0.1

Supplementary Notes

Supplementary Notes 1

Building on the baseline scenario and incorporating five key CO_2 reduction strategies, we developed two additional scenarios: the regulatory scenario and the blueprint scenario. These scenarios differ based on the maturity and implementation intensity of the technological solutions employed. Both scenarios are designed to align with China's energy conservation and emission reduction targets.

Consequently, the technical parameters used in this study were drawn from government policy documents and supplemented by relevant literature. The primary difference between these two scenarios lies in the specific emission reduction measures adopted, as well as variations in macroeconomic factors such as total population and GDP, which are integrated into the model. These distinctions lead to differing outcomes in terms of emission reduction effectiveness. The definitions and key distinctions among the three scenarios are as follows:

- The baseline scenario refers to the current development scenario, which takes into account various policy measures that have been issued based on the current level of social and economic development in China. The macroeconomic indicators such as the population and GDP in China continue to develop according to the current situation. The energy consumption intensity and level of public buildings are determined by the energy-saving technology level of buildings driven by China's future socio-economic development. In this scenario, the CO_2 emissions from energy use during the operation phase of buildings in Nanjing will reach 5325.45 kt CO_2e by 2050.
- The regulatory scenario is positioned towards green and sustainable development, promoting the design and energy-saving renovation of public buildings in China, and completing energy-saving renovation work of large public buildings with renovation value as soon as possible. The total population and GDP of China have increased at a moderate rate, and the industrial structure has been optimized. The energy consumption intensity and level have decreased compared to the baseline scenario. Under these settings, the CO_2 emissions from energy use during the operation phase of buildings in Nanjing will be 3225.55 kt CO_2e.
- The blueprint scenario is the optimization scenario, which is based on achieving the "carbon neutrality" goal and further expanding the development of energy-saving technologies. The GDP growth rate has decreased and is even lower than the first two scenarios. The total population in China has reached its peak ahead of schedule and has shown a downward trend. The energy use structure has been optimized, and the energy consumption intensity and CO_2 emissions of public buildings have significantly decreased. In this scenario, CO_2 emissions will decrease to 2055.29 kt CO_2e by 2050.

Supplementary Notes 2

This work integrates both macro and micro-level data from device-based sources(Supplementary Tables), developing baseline, regulatory, and blueprint scenarios to forecast future energy demand and CO_2 emissions during the operational phase of buildings. Based on these scenarios, we incorporate relevant emission reduction technologies from both the demand and supply sides to analyze potential emission reduction pathways, with a focus on differentiated strategies for various street types. For "Commercial and Industrial building-base streets," which are dominated by manufacturing and trade services, there is a need to enhance technological innovation to meet higher emission reduction targets. This includes improving the operational efficiency of end-use energy equipment to lower the CO_2 emission intensity of commercial and service buildings. In contrast, "Life service building-base streets," characterized by high-value-added consumer services and predominantly residential buildings, should prioritize accelerating the electrification of residential energy use. For example, adopting advanced cooking technologies such as induction cookers and microwaves to replace traditional coal-fired and biomass stoves can significantly reduce emissions[15]. Overall, it is essential to systematically evaluate the emission reduction priorities of

different streets based on their economic development characteristics, functional roles, architectural features, and other relevant factors. This approach enables the formulation of differentiated and customized emission reduction strategies tailored to the unique needs of each area.

In addition to reducing emissions in the energy-consuming sectors on the demand-side, the implementation of supply-side emission reduction technologies is crucial for decarbonizing the operational phase of buildings (Supplementary Fig.2). Firstly, to meet climate targets, it is imperative to gradually phase out coal-fired power plants, which are major sources of emissions[16]. Our findings suggest that promoting the early retirement of these coal-fired power plants can significantly alleviate future emission reduction pressures in the building sector and substantially decrease cumulative CO_2 emissions from coal-based power generation. For instance, under the blueprint scenario, delaying the retirement of coal-fired power plants by just 10 years would result in an additional 158,406.4 kt CO_2e. Secondly, transforming the energy structure on the power generation side is essential for decarbonization. This involves significantly increasing the share of zero-carbon energy sources, such as wind power, photovoltaics[17,18], and nuclear power. Additionally, the deployment of zero-emission power plants, such as coal-biomass combined combustion power plants (CBECCS), should be accelerated[19,20]. Our study shows that as existing coal-fired power plants are retired, the integration of zero-carbon energy plants will yield substantial emission reduction benefits. In the blueprint scenario, solar energy, wind energy, and CBECCS are projected to constitute 48.56%, 29.13%, and 8.09% of the power supply structure by 2050. Furthermore, improving the efficiency of existing coal-fired and gas-fired power plants, as well as other power generation units, will contribute significantly to reducing CO_2 emissions.

Supplementary Reference

[1] Cui, R. *et al.* A plant-by-plant strategy for high-ambition coal power phaseout in China. *Nature Communications* **12**, 10, doi:10.1038/s41467-021-21786-0 (2021).

[2] Ma, M. Y. *et al.* Roadmap towards clean and low-carbon heating to 2060: The case of northern urban region in China. *Energy* **284**, doi:10.1016/j.energy.2023.129181 (2023).

[3] Rodrigues, C., Rodrigues, E., Fernandes, M. S. & Tadeu, S. Prospective life cycle approach to buildings' adaptation for future climate and decarbonization scenarios. *Applied Energy* **372**, doi:10.1016/j.apenergy.2024.123867 (2024).

[4] Shi, J. C., Chen, W. Y. & Yin, X. Modelling building's decarbonization with application of China TIMES model. *Applied Energy* **162**, 1303-1312, doi:10.1016/j.apenergy.2015.06.056 (2016).

[5] Aguacil, S., Duque, S., Lufkin, S. & Rey, E. Designing with building-integrated photovoltaics (BIPV): A pathway to decarbonize residential buildings. *Journal of Building Engineering* **96**, doi:10.1016/j.jobe.2024.110486 (2024).

[6] Sarica, K., Harputlugil, G. U., Inaner, G. & Kollugil, E. T. Building sector emission reduction assessment from a developing European economy: A bottom-up modelling approach. *Energy Policy* **174**, doi:10.1016/j.enpol.2023.113429 (2023).

[7] Kong, X. F., Lu, S. L. & Wu, Y. A review of building energy efficiency in China during "Eleventh Five-Year Plan" period. *Energy Policy* **41**, 624-635,

doi:10.1016/j.enpol.2011.11.024 (2012).
[8] Delmastro, C., Lavagno, E. & Mutani, G. Chinese residential energy demand: Scenarios to 2030 and policies implication. *Energy and Buildings* **89**, 49-60, doi:10.1016/j.enbuild.2014.12.004 (2015).
[9] Langevin, J. *et al.* Demand-side solutions in the US building sector could achieve deep emissions reductions and avoid over $100 billion in power sector costs. *One Earth* **6**, 1005-1031, doi:10.1016/j.oneear.2023.07.008 (2023).
[10] Kannan, R. & Strachan, N. Modelling the UK residential energy sector under long-term decarbonisation scenarios: Comparison between energy systems and sectoral modelling approaches. *Applied Energy* **86**, 416-428, doi:10.1016/j.apenergy.2008.08.005 (2009).
[11] Gambhir, A. *et al.* A hybrid modelling approach to develop scenarios for China's carbon dioxide emissions to 2050. *Energy Policy* **59**, 614-632, doi:10.1016/j.enpol.2013.04.022 (2013).
[12] McNeil, M. A. *et al.* Energy efficiency outlook in China's urban buildings sector through 2030. *Energy Policy* **97**, 532-539, doi:10.1016/j.enpol.2016.07.033 (2016).
[13] Xiao, K., Yu, B. L., Cheng, L., Li, F. & Fang, D. B. The effects of CCUS combined with renewable energy penetration under the carbon peak by an SD-CGE model: Evidence from China. *Applied Energy* **321**, doi:10.1016/j.apenergy.2022.119396 (2022).
[14] de Oliveira, L. P. N. *et al.* Critical technologies for sustainable energy development in Brazil: technological foresight based on scenario modelling. *Journal of Cleaner Production* **130**, 12-24, doi:10.1016/j.jclepro.2016.03.010 (2016).
[15] Tang, B. J., Guo, Y. Y., Yu, B. Y. & Harvey, L. D. D. Pathways for decarbonizing China's building sector under global warming thresholds. *Applied Energy* **298**, 15, doi:10.1016/j.apenergy.2021.117213 (2021).
[16] Jakob, M. *et al.* The future of coal in a carbon-constrained climate. *Nature Climate Change* **10**, 704-707, doi:10.1038/s41558-020-0866-1 (2020).
[17] Skandalos, N. & Karamanis, D. An optimization approach to photovoltaic building integration towards low energy buildings in different climate zones. *Applied Energy* **295**, 24, doi:10.1016/j.apenergy.2021.117017 (2021).
[18] Zhang, K. *et al.* Model predictive control for demand flexibility: Real-world operation of a commercial building with photovoltaic and battery systems. *Advances in Applied Energy* **7**, 16, doi:10.1016/j.adapen.2022.100099 (2022).
[19] Xing, X. F. *et al.* Spatially explicit analysis identifies significant potential for bioenergy with carbon capture and storage in China. *Nature Communications* **12**, 12, doi:10.1038/s41467-021-23282-x (2021).
[20] Fan, J. L. *et al.* Co-firing plants with retrofitted carbon capture and storage for power-sector emissions mitigation. *Nature Climate Change* **13**, 807-+, doi:10.1038/s41558-023-01736-y (2023).
